# Portable Bio/Chemosensoristic Devices: Innovative Systems for Environmental Health and Food Safety Diagnostics

**DOI:** 10.3389/fpubh.2017.00080

**Published:** 2017-05-05

**Authors:** Roberto Dragone, Gerardo Grasso, Michele Muccini, Stefano Toffanin

**Affiliations:** ^1^Institute of Nanostructured Materials (ISMN), Consiglio Nazionale delle Ricerche (CNR), Rome, Italy; ^2^Institute of Nanostructured Materials (ISMN), Consiglio Nazionale delle Ricerche (CNR), Bologna, Italy

**Keywords:** agro-food supply chain, milk, on-site diagnostics, electroanalytical methods, biosensoristic devices, surface plasmon resonance, lab-on-a-chip

## Abstract

This mini-review covers the newly developed biosensoristic and chemosensoristic devices described in recent literature for detection of contaminants in both environmental and food real matrices. Current needs in environmental and food surveillance of contaminants require new simplified, sensitive systems, which are portable and allow for rapid and on-site monitoring and diagnostics. Here, we focus on optical and electrochemical bio/chemosensoristic devices as promising tools with interesting analytical features that can be potentially exploited for innovative on-site and real-time applications for diagnostics and monitoring of environmental and food matrices (e.g., agricultural waters and milk). In near future, suitably developed and implemented bio/chemosensoristic devices will be a new and modern technological solution for the identification of new quality and safety marker indexes as well as for a more proper and complete characterization of abovementioned environmental and food matrices. Integrated bio/chemosensoristic devices can also allow an “holistic approach” that may prove to be more suitable for diagnostics of environmental and food real matrices, where the copresence of more bioactive substances is frequent. Therefore, this approach can be focused on the determination of net effect (mixture effect) of bioactive substances present in real matrices.

## Environmental Health and Food Safety: Scenario and Needs

Over the last few years, the abiotic contaminants levels in the environmental compartments and food increased to the point where they can cause potential human health effects due to exposure to chemical toxic substances. In particular, the interactions between environment and food supply chain that mainly occur at primary production level (including harvesting, milking and farmed animal production prior to slaughter, hunting and fishing, and harvesting of wild products) can cause serious both short- and long-term detrimental effects on human health.

Environmental and food safety remains a major global challenge, in particular in developing countries, where socioeconomic status predisposes a large share of the population to a direct environmental-origin contamination and/or consumption of contaminated food products.

To minimize the negative and dramatic impacts (especially in developing countries) of chemical toxic substances of anthropic origin on environmental and human health, several focused actions are needed. For instance, promoting a sustainable use of chemicals and agrochemicals (e.g., pesticides, veterinary antibiotics, and food additives); the development of toxicovigilance practices and systems (1) and more effective primary prevention strategies (raising users’ awareness and promoting the use of good practice codes); moreover, implementation of legislative regulations; and the development of proper infrastructures and effective protocols for safely recycle and dispose of hazardous wastes are necessary.

Against this background, such critical issues have produced a great demand for simplified, sensitive, and rapid screening methods (2, 3), without (or with reduced) sample pretreatments, suitable for environmental monitoring and surveillance at critical control points throughout the entire agro-food supply chain. In fact, recent progress and challenges in the field of analytical chemistry are focused on improving analytical methods with reduced environmental impact and developing new analytical sensoristic devices for continuous monitoring and diagnostics oriented toward environmental health and food safety.

On-site, cost-effective sensoristic devices capable of routine, sensitive, and selective detection of a range of targeted contaminants present in the environment and foods can be employed, for instance, to overcome time limitations and to reduce costs of sample collection and transport to laboratories, thus providing benefits for a rapid diagnostics and early corrective actions.

## Emerging Role of Portable Bio/Chemosensoristic Devices in Environment and Agro-Food Supply Chain Monitoring

For on-site diagnostics and environmental/food monitoring purposes, the application of standard and traditional analytical techniques (very sensitive and selective techniques, but costly, time consuming and requiring trained personnel with technical skills to perform the analysis) is in contrast with the current need of rapid, cheap, easy-to-use, and portable devices (4).

For these purposes, chemosensoristic and biosensoristic devices (herein collectively referred to as bio/chemosensoristic devices) are promising tools with interesting analytical features, which can be potentially exploited for on-site real-time applications, diagnostics, and screening for both environmental and food matrices. Such devices could be employed, e.g., to overcome existing limitations in measurements currently used in environmental and agro-food fields. While those measurements are mainly focused on the independent analyses of various parameters and analytes, complexity of environmental and food matrices requires a new holistic-like approach (4).

For instance, regarding nutritional and toxicology characterization of foods, a broader modern vision based on the concept of “whole food” is taking off (4, 5). Environmental and food matrices are complex mixtures of bioactive molecules, whose complex interactions between individual components could eventually produce different and hardly theoretically predictable “net effects.” “Net effect” is necessarily different from single effects of each individual substance, and it could be additive (when it is equal to the sum of contributions of individual substances), synergistic (when it is greater than the sum of contributions of the individual substances), and antagonistic (when it is less than the sum of contributions of individual substances).

For a more proper and complete characterization of food matrices (and environmental ones) “as a whole,” integrated analyses of physical, chemical, and biological parameters through sensoristic devices could be more suitable. The multichannel platform BEST (6) is a HACCP-like monitoring system that follows this approach for the generation of integrated analytical information. More specifically, BEST focuses on identification, control, simultaneous, and non-stop monitoring of anomalous variations throughout agro-zootechnical productions, developed to allow simultaneous collection and analysis of multiple signals. Such signals are produced from a battery of selected analogical and/or digital bio/chemosensoristic devices (or probes), integrated with each other and functioning simultaneously. The simultaneous acquisition of multiparameters and integrated information can be useful in determining correlations and relationships among different data (through multivariate data analysis), and it can constitute a flexible grid of indexes and multiple markers in series. Such integrated analytical approach helps to define a “fingerprint” and to identify new marker indexes of food matrices. A field validation of BEST prototype is taking place in a farm in the Lazio region (Italy) within project ALERT (7). This project, funded by the Italian Ministry of Economic Development under the Call Industria 2015 New technologies for Made in Italy (www.alert2015.it), aims at developing the BEST prototype for industrial-scale production. Another new interesting approach for innovative monitoring and diagnostics of the environment and the agro-food supply chain is provided by a recent patented physicochemical sensing device called SNOOP (8). SNOOP is a multiparameter and multisignal sensoristic device that uses advanced and appropriately designed sensitive materials. Such sensitive materials can be both biological materials (e.g., whole cells, enzymes, and aptamers) and chemical materials (newly synthesized and/or functionalized inorganic and organic materials), whose one of the main features is the specific interaction with the target analyte(s) present in real and complex matrices. Such interactions can produce specific or aspecific physicochemical (electric or optic) responses, and the simultaneous use of different sensitive materials and the combination/integration of outgoing signals can significantly increase the screening ability of SNOOP.

### Electrochemical Bio/Chemosensoristic Devices

The field of electrochemical and optical bio/chemosensoristic devices has grown rapidly in the past few years. Thanks to advantages provided by intrinsic analytical features and the development of new advanced sensitive materials, the employment of these devices has proved to be very useful for chemical contaminants detection in environmental and food matrices (Tables S1 and S2 in Supplementary Material). In particular, biosensors or biosensoristic devices (*an integrated receptor–transducer device, which is capable of providing selective quantitative or semiquantitative analytical information using a biological recognition element*) (9) hold promise to be relatively cheap and portable devices for *in situ* detection of environmental and food contaminants (10, 11).

A recent review on key research interests in the development of biosensors in South Africa has highlight a particular interest on the development of electrochemical (amperometric, impedimetric, potentiometric, and voltammetric) biosensor due to low fabrication and analytical equipment costs, in particular, for pesticides and heavy metals detection. Other research areas include nanotechnology, identification and validation of biomarkers, and development of biorecognition agents (antibodies and aptamers) and new biosensor design approaches (e.g., development of new materials) (12).

In recent literature, enzymes (13–15) and whole cells (16) seem to have been replaced with antibodies (17–23) and aptamers (24–29) as recognition elements in electrochemical and optical biosensoristic devices.

Regarding electrochemical devices, they possess unique features to address the challenges of field and on-site analytical chemistry: possibility of miniaturization and portability, sensitivity, selectivity, a wide linear range of detection, minimal power requirement, and cost-effective instrumentation. Voltammetry is one of the most widely used electroanalytical techniques for electrochemical detection in bio/chemosensoric devices (see Tables S1 and S2 in Supplementary Material). In fact, various voltammetric techniques possess intrinsic analytical advantages and features and included excellent sensitivity, rapid analysis times, and possibility of simultaneous determination of different analytes. In voltammetric pulse techniques, through different modulation of the applied potential, a higher speed of measurement and sensitivity (useful for determination of species at trace levels) can be achieved. In particular, differential pulse voltammetry and square-wave voltammetry have been extensively described in the recent literature for detection of various chemical contaminants in environmental samples (24, 25, 30–35). Other widely used electrochemical techniques includes cyclic voltammetry (for studies on redox behavior of analytes) (31, 36, 37) and stripping techniques (characterized by preconcentration step of the analyte onto or into the working electrode to achieve a greater sensitivity) (38–42). In addition, the latter are commonly applied for determination of metal speciation (chemical form can influence bioavailability of metals) useful for environmental risk assessment of metal pollution (43). Amperometry is another widely used electrochemical technique in bio/chemosensoristics (13–17, 44–51). Together with voltammetric techniques, electrochemical impedance spectroscopy is an extremely useful technique for a broad range of applications, including characterization of materials and detecting interaction between recognition elements (e.g., antibodies and aptamers) of sensoristic devices and analyte, through measures of changes in electrical surface properties of electrodes (26, 28). To improve analytical features and performances of electrochemical techniques, the last decades have witnessed a tremendous development of innovative sensitive materials for surface functionalization of electrodes. Several advances in the development of bio/chemosensors (in particular for electrochemical devices) have been achieved through the employment of (modified) electrodes (Tables S1 and S2 in Supplementary Material). Traditional mercury-based electrodes (39, 41) have gradually been replaced (because of low mechanical stability and toxicity of mercury) by other electrodes made of better suitable materials. As replacement of mercury, alternative materials (with similar or better analytical features) have been employed and/or developed: bismuth (a non-toxic element with high hydrogen overpotential and good mechanical stability) (52), boron-doped diamond (with a wider electrochemical potential window and reduced fouling compared to traditional materials) (34), nitrogen-doped graphene (doping converts an excellent conductor as graphene into a p- or n-type semiconductor) (49), and single and multiwalled carbon nanotubes and nanoparticles. Looking at recent literature, a considerable attention has been paid to the development and exploitation of nanostructured materials (nanoparticles, nanowires, or nanotubes) for sensoristic purposes: carbon-based (e.g., single-walled and multiwalled carbon nanotubes) (17, 31, 35, 42, 45, 50) and nanoparticles with different chemical composition (13, 24, 26–30, 32, 40, 50, 51). These nanomaterials (also functionalized) can modify surface architectures and functions of electrodes by, for instance, (i) enlarging active surface (e.g., increasing of docking sites for biological recognition elements) and (ii) enhancing electron transfer or electrical properties and amplify signals in general. Another interesting supramolecular-based approach to develop innovative materials for electrode modification is the synthesis of molecular and ion imprinted polymers. These are synthetic polymers able to mimicking biological recognition elements, like antibodies and aptamers, useful for the design of high-specificity sensoristic devices (30, 35, 38, 50, 53). Basically, these polymers are obtained from a copolymerization process of suitable monomers in the presence of a molecular or ionic template (the target analyte); the successive removal of the template leaves in the polymer structure binding sites that can re-host the analyte. Although they bring several advantages in terms of durability and cost-effective production (compared to aptamers and antibodies), it is still necessary to solve some problems related to heterogeneity of binding sites that can bring to non-specific bindings.

### Optical Biosensoristic Devices and Lab-on-a-Chip (LOC)

The recent interest in optical biosensoristic devices for food analysis, with fluorescent, bioluminescent or chemiluminescent labels for detection, as well as the direct (label-free) detection (i.e., no reporter elements to generate a signal are needed) (54, 55), is increasing. The development of label-free technologies and in particular label-free surface plasmon resonance (SPR) has become the greatest example of employment of the technology as a routine analytical method in such fields (56).

Actually, biosensoristic devices based on SPR are ideal platforms for the label-free detection of molecular monolayers as they allow for qualitative and quantitative multiplexing measurements of biomolecular interactions in real-time without requiring a labeling procedure in the framework of food safety (57, 58).

Indeed, by using SPR-based immunosensors, one can obtain robust and quantitative results with narrow- or broad-spectrum specificity in relatively short time. In the case of milk, SPR circumvents the issues related to turbidity and protein fouling (both are generally limiting factors for optical-based biosensoristic devices application for milk testing) by measuring the refractive index modulation on the reverse side of the metal film where the biological selective element is immobilized (18, 59).

Since late 90s, SPR biosensoristic devices have become the main tool for the study of biomolecular interactions in life science, with successful applications in the field of food safety (58).

Although there are great advantages of the SPR technology, some disadvantages are evident: high cost of the readout instrumentation and a still high cost of the consumables (sensing chip and reagents) and large instrumentation footprint.

In recent years, nanoplasmonics (e.g., noble metal nanoparticles, nanometallic gratings, or a combination of metallic nanocavities organized in nanogratings) has shown a great potential in overcoming the technological/commercial limits of SPR (60) and for developing nanoplasmonic detection platforms. Even though important technological effort is still to be done to be competitive with point-of-care screening technologies, the integration on the same disposable and miniaturized platform of low-cost photonics devices with multiplexing nanoplasmonic and advanced microfluidics system can be considered as new and non-disruptive technology able to ensure competitiveness from both the economic and the detection point of views.

The development of advanced photonic biosensoristic devices has to be brought beyond the state of the art of the point-of-care-diagnostic systems by the synergetic integration of the different technological building blocks with consequent improvement of the single-component outputs. Moreover, the introduction of outperforming light-excitation/detection scheme allows for unraveling the potentiality of the sensor in terms for disposability, reliability, miniaturization, and multiplexing while providing laboratory quality analysis (61).

Within the current point-of-care diagnostic market, there is a limited number of systems that operate without the requirement for a dedicated desktop reader, and there are no quantitative, portable diagnostic platforms with multiple detection methods. The components from existing laboratory equipment are too bulky, fragile, and expensive and require too much mechanical integration to be consolidated into a point-of-care device (55).

Miniaturization (from microelectrodes/nanosensors to microfluidic platforms) is an increasing trend as a response to these needs to develop new miniature and portable analytical devices for environmental and food monitoring and diagnostics.

In this scenario, LOC devices have shown themselves to be highly effective for laboratory-based research, where their superior analytical performance has established them as efficient tools for complex tasks and a promising tools for a number of environmental monitoring applications, i.e., continuous surveillance of selected parameters and contaminant concentrations (62) and for agricultural and food safety (63). Referring to the state of the art in the recently developed LOC methods (64), it can be observed that they are based on nucleic acid amplification, biosensoristic devices, flow cytometry, spectrometry techniques, and multisensors systems.

However, to date, they have not been well suited to point-of-care or in-the-field applications: although the chips themselves are cheap and small, they must generally be used in conjunction with bulky optical detectors, which are needed to identify or quantify the analytes or reagents present. Furthermore, most existing detectors are limited to analysis of a single analyte at a predetermined location on the chip. The lack of an integrated, multiplexing, and fast detection scheme (one which is miniaturized, integrated, and able to monitor multiple locations on the chip) is a major obstacle to the deployment of diagnostic devices in the field. This issue has prevented the development of more complex tests where rapid, kinetic, or multipoint analysis is required.

## Conclusion

Development of improved electrochemical and optical bio/chemosensoristic devices represents a technological challenge to broaden boundaries of field diagnostics and monitoring environmental and food samples. In particular, specific improved features of integration, portability (e.g., sensors equipped with built-in reading systems), cheapness, simplification of experimental protocols (less time- and labor-demanding protocols), and development of efficient high-throughput approaches are required. Concerning LOC devices, fast detection scheme and the ability to monitor at multiple locations on the chip could ensure a high selectivity and sensitivity for the analyte of interest. All these devices could be employed for the identification of new quality and safety marker indexes in real matrices as well as for the determination of mixture effects of bioactive substances.

## Author Contributions

All authors have made equal contributions in the writing and revising of this mini-review.

## Conflict of Interest Statement

The authors declare that the research was conducted in the absence of any commercial or financial relationships that could be construed as a potential conflict of interest.
