# Supplementary material for: Portable Bio/Chemosensoristic Devices: Innovative Systems for Environmental Health and Food Safety Diagnostics
Source: Front Public Health. 2017 May 5;5:80. doi: 10.3389/fpubh.2017.00080 (PMC5418341; doi:10.3389/fpubh.2017.00080)
Supplement: Supplementary file 1 [file Table_1.PDF]

Supplementary Material

Portable bio/chemosensoristic devices: innovative systems for environmental health and food safety diagnostics

Roberto Dragone, Gerardo Grasso*, Michele Muccini, Stefano Toffanin

*** Correspondence:** Dr. Gerardo Grasso: gerardo.grasso@ismn.cnr.it

Table S1. Bio/chemosensoristic devices for environmental samples analysis

Sensoristic device	Detection technique	Sensitive material	Analyte	Matrix	Advantages and disadvantages	Linear range (μmol L^{-1})	LoD (μmol L^{-1})	Reference
Electrochemical chemosensor	differential pulse stripping voltammetry	ion imprinted polymer	Pb(II)	tap water river water salt water wastewater	less interference effect (e.g. in presence of Cu(II)) and lower LoD compared to other Pb(II) voltammetric sensors; disadvantages not described	0.001-0.008	0.0006	Alizadeh and Amjadi, 2011
	square wave anodic stripping voltammetry	bismuth nanoparticle-porous carbon paste	Cd(II)	tap water ground water polluted waters	sample dilution is necessary to minimize matrix effects; interferences from other divalent metal cations or high content of organic matter (for Ni(II)	0 – 0.9	0.007	Niu et al., 2015

	square wave adsorptive cathodic stripping voltammetry (for Ni(II))		Pb(II)		detection); Cu(II) interfere with Cd(II) detection	0 – 0.5	0.0031	
			Ni(II)			0.2 – 2.6	0.93	
	differential pulse voltammetry	silver nanoparticles decorated with reduced graphene oxide modified glassy carbon	nitrobenzene	waste water	good selectivity (low interferences by other nitroaromatic compounds and metal ions) and storage stability; disadvantages not described	500- 9.0 x 10^5	300	Karuppiah et al., 2015
	square wave voltammetry	boron-doped diamond electrode	atrazine	river water	low residual current; interference by Zn(II)	0.05 - 40	0.01	Švorc et al., 2013
	square wave voltammetry	molecularly imprinted polymer +carboxyl-functionalized multi-walled carbon nanotubes	diuron	river water	high selectivity; absence of matrix effects; disadvantages not described	0.052 - 1.3	0.009	Wong et al., 2015
	adsorptive stripping differential pulse voltammetry	multi-walled carbon nanotubes functionalized with carboxyl groups + graphene oxide	tetracycline	river water	no sample pretreatment; highly selective; disadvantages not described	20 - 310	0.36	Wong et al., 2015b
	square wave voltammetry	fullerene (C_{60})	bisphenol A	wastewater	long-term stability; no interferences from other similar compounds; filtration (Whatman filter paper) of samples is needed	0.074 - 0.23	0.0037	Rather et al., 2013

	amperometry	nitrogen-doped graphene sheets and chitosan		river water	no interferences from organic compound and ions; no sample pretreatment; disadvantages not described	0.01 - 1.3	0.005	Fan et al., 2012
	amperometry	molecular imprinted polymer +multi-walled carbon nanotubes and gold nanoparticles		tap water	high selectivity; disadvantages not described	0.11 - 8200	0.0036	Huang et al., 2011
	cyclic voltammetry and square wave voltammetry	nylon 6,6/ multi-walled carbon nanotubes /Fe_3O_4 nanofibers modified glassy carbon	lindane	drinking water	no interferences from inorganic ions; 60:40 (v/v) methanol-sample mixture and tetrabutyl-ammonium bromide are required	9.9×10^{-6} - 5	0.032	Fayemi et al., 2016
	differential pulse voltammetry	molecularly imprinted polymer +nickel nanoparticles-graphene	tetrabromo-bisphenol A	tap water<br>rain<br>lake water	good selectivity; sample filtration is required; after filtration (0.45 µm membrane), the samples were prepared with PBS buffer (pH 7.0)	0.0005-10	0.00013	Chen et al., 2014
	cyclic voltammetry	wax patterning, paper chemically modified molybdate ions	phosphate	river water	no matrix effect was observed; disadvantages not described	0 - 300	4	Cinti et al., 2016

Optical chemosensor	radiometry	nitrate ionophore-based membrane	nitrate	tap water<br>river water<br>sea water<br>well water	short analysis time (few minutes); no sensor conditioning is needed; equilibration time is higher and the accuracy is lower (compared to ion selective electrodes)	2 - 1× 10^{6}	/	Fernández-Ramos et al., 2008
Electrochemical biosensor	amperometry	anti-atrazine antibodies + single-walled carbon nanotubes	atrazine	sea water<br>river water	short analysis time (15 min.); higher cross-reactivity to two atrazine degradation products	4.63× 10^{-6} - 0.0463	4.6 × 10^{-6}	Belkhamssa et al., 2016
	electrochemical impedance spectroscopy	anti-acetamiprid aptamers + gold nanoparticles	acetamiprid	wastewater	sample filtering is required; high selectivity; analysis time 3 h	0.005 - 0.6	0.001	Fan et al., 2013
	photo-electrochemistry	anti-17β-estradiol aptamer + cadmium selenide -modified titanium dioxide nanotube arrays	17β-estradiol	medical wastewater<br>lake water<br>tap water	sample filtering is required; high selectivity and affinity; disadvantages not described	5 × 10^{-7}- 0.00015	3.3× 10^{-8}	Fan et al., 2014
	square wave voltammetry	anti-Cu(II) aptamers + gold nanoparticles	Cu(II)	lake water	no interferences from other divalent cations; short time analysis (1 min); disadvantages not described	0.001- 0.01	/	Chen et al., 2011
	amperometry	acetylcholinesterase	As(III)	tap water	good storage stability (15 days at 4 °C in buffer solution); interferences from Ni(II) and Cu(II)	0.01 - 0.1	0.011	Sanllorente-Méndez et al., 2010

	amperometry	*E. coli* reporter strain		groundwater	relative short analysis time (25 to 50 min) compared with other similar methods; pH adjustment (neutralization) of samples required	0.0125 - 0.4	/	Cortés-Salazar et al., 2013
	differential pulse voltammetry	anti-As(III) aptamers adsorbed on polydiallyldi-methyl-ammonium		tap water<br>lake water	label-free; no interferences from metal ions; filtration and boiling (15 min.) of samples are required	0.0002 - 0.1	0.00015	Cui et al., 2016
Optical biosensor	surface plasmon resonance	anti- carbaryl antibodies	carbaryl	groundwater	no sample pretreatment are required; disadvantages not described	0.00099 - 0.099	0.0068	Mauriz et al., 2006
				tap water			0.0075	
				river water			0.0077	

REFERENCES

Alizadeh T, Amjadi S. Preparation of nano-sized Pb^{2+} imprinted polymer and its application as the chemical interface of an electrochemical sensor for toxic lead determination in different real samples. *J Hazard Mater* (2011) 190(1):451–9. doi:10.1016/j.jhazmat.2011.03.067

Belkhamssa N, Justino CI, Santos PS, Cardoso S, Lopes I, Duarte AC, et al. Label-free disposable immunosensor for detection of atrazine. *Talanta* (2016) 146:430–4. doi:10.1016/j.talanta.2015.09.015

Chen Z, Li L, Mu X, Zhao H, Guo L. Electrochemical aptasensor for detection of copper based on a reagentless signal-on architecture and amplification by gold nanoparticles. *Talanta* (2011) 85:730–5. doi:10.1016/j.talanta.2011.04.056

Chen H, Zhang Z, Cai R, Rao W, Long F. Molecularly imprinted electrochemical sensor based on nickel nanoparticles-graphene nanocomposites modified electrode for determination of tetrabromobisphenol A. *Electrochim Acta* (2014) 117:385–92. doi:10.1016/j.electacta.2013.11.185

Cinti S, Talarico D, Palleschi G, Moscone D, Arduini F. Novel reagentless paper-based screen-printed electrochemical sensor to detect phosphate. *Anal Chim Acta* (2016) 919:78–84. doi:10.1016/j.aca.2016.03.011

Cortés-Salazar F, Beggah S, van der Meer JR, Girault HH. Electrochemical As(III) whole-cell based biochip sensor. *Biosens Bioelectron* (2013) 47:237–42. doi:10.1016/j.bios.2013.03.011

Cui L, Wu J, Ju H. Label-free signal-on aptasensor for sensitive electrochemical detection of arsenite. *Biosens Bioelectron* (2016) 79:861–5. doi:10.1016/j.bios.2016.01.010

Fan H, Li Y, Wu D, Ma H, Mao K, Fan D, et al. Electrochemical bisphenol A sensor based on N-doped graphene sheets. *Anal Chim Acta* (2012) 711:24–8. doi:10.1016/j.aca.2011.10.051

Fan L, Zhao G, Shi H, Liu M, Li Z. A highly selective electrochemical impedance spectroscopy-based aptasensor for sensitive detection of acetamiprid. *Biosens Bioelectron* (2013) 43:12–8. doi:10.1016/j.bios.2012.11.033

Fan L, Zhao G, Shi H, Liu M, Wang Y, Ke H. A femtomolar level and highly selective 17β-estradiol photoelectrochemical aptasensor applied in environmental water samples analysis. *Environ Sci Technol* (2014) 48(10):5754–61.doi:10.1021/es405685y

Fayemi OE, Adekunle AS, Ebenso EE. A sensor for the determination of lindane using PANI/Zn, Fe (III) oxides and nylon 6, 6/MWCNT/Zn, Fe (III) oxides nanofibers modified glassy carbon electrode. *J Nanomater* (2016) 2016, 1-10.doi:10.1155/2016/4049730

Fernández-Ramos MD, Greluk M, Palma AJ, Arroyo-Guerrero E, Gómez-Sánchez J, Capitán-Vallvey LF. The use of one-shot sensors with a dedicated portable electronic radiometer for nitrate measurements in aqueous solutions. *Meas Sci Technol* (2008) 19(9):095204. doi:10.1088/0957-0233/19/9/095204

Huang J, Zhang X, Lin Q, He X, Xing X, Huai H, et al. Electrochemical sensor based on imprinted sol-gel and nanomaterials for sensitive determination of bisphenol A. *Food Control* (2011) 22(5):786–91. doi:10.1016/j.foodcont.2010.11.017

Karuppiah C, Muthupandi K, Chen SM, Ali MA, Palanisamy S, Rajan A, et al. Green synthesized silver nanoparticles decorated on reduced graphene oxide for enhanced electrochemical sensing of nitrobenzene in waste water samples. *RSC Adv* (2015) 5(39):31139–46. doi:10.1039/C5RA00992H

Mauriz E, Calle A, Abad A, Montoya A, Hildebrandt A, Barceló D, et al. Determination of carbaryl in natural water samples by a surface plasmon resonance flow-through immunosensor. *Biosens Bioelectron* (2006) 21(11):2129–36. doi:10.1016/j.bios.2005.10.013

Niu P, Fernández-Sánchez C, Gich M, Ayora C, Roig A. Electroanalytical assessment of heavy metals in waters with bismuth nanoparticle-porous carbon paste electrodes. *Electrochim Acta* (2015) 165:155–61. doi:10.1016/j.electacta.2015.03.001

Rather JA, De Wael K. Fullerene-C 60 sensor for ultra-high sensitive detection of bisphenol-A and its treatment by green technology. Sens Actuators B Chem (2013) 176:110–7. doi:10.1016/j.snb.2012.08.081

Sanllorente-Méndez S, Domínguez-Renedo O, Arcos-Martínez MJ. Immobilization of acetylcholinesterase on screen-printed electrodes. Application to the determination of arsenic (III). *Sensors (Basel)* (2010) 10(3):2119–28. doi:10.3390/s100302119

Švorc Ľ, Rievaj M, Bustin D. Green electrochemical sensor for environmental monitoring of pesticides: determination of atrazine in river waters using a boron-doped diamond electrode. *Sens Actuators B Chem* (2013) 181:294–300.doi:10.1016/j.snb.2013.02.036

Wong A, Foguel MV, Khan S, de Oliveira FM, Tarley CRT, Sotomayor MD. Development of an electrochemical sensor modified with MWCNT-COOH and MIP for detection of diuron. *Electrochim Acta* (2015) 182:122–30. doi:10.1016/j.electacta.2015.09.054

Wong A, Scontri M, Materon EM, Lanza MR, Sotomayor MD. Development and application of an electrochemical sensor modified with multi-walled carbon nanotubes and graphene oxide for the sensitive and selective detection of tetracycline. *J Electroanal Chem* (2015b) 757:250–7. doi:10.1016/j. jelechem.2015.10.001
