# Supplementary material for: Portable Bio/Chemosensoristic Devices: Innovative Systems for Environmental Health and Food Safety Diagnostics
Source: Front Public Health. 2017 May 5;5:80. doi: 10.3389/fpubh.2017.00080 (PMC5418341; doi:10.3389/fpubh.2017.00080)
Supplement: Supplementary file 2 [file Table_2.PDF]

Supplementary Material

Portable bio/chemosensoristic devices: innovative systems for environmental health and food safety diagnostics

Roberto Dragone, Gerardo Grasso*, Michele Muccini, Stefano Toffanin

*** Correspondence:** Dr. Gerardo Grasso: gerardo.grasso@ismn.cnr.it

Table S2. Bio/chemosensoristic devices for food samples analysis

Sensoristic device	Detection technique	Sensitive material	Analyte	Matrix	Advantages and disadvantages	Linear range (μmol L^{-1})	LoD (μmol L^{-1})	Reference
Electrochemical chemosensor	anodic stripping voltammetry	hanging mercury drop	Pb(II)	raw milk	reduced intermetallic interferences; environmental concerns related to the toxicity of mercury; sample digestion is required	0.0024 - 0.98	0.0012	Shahbazi et al., 2016
			Cd(II)			0.0027 - 2.0	0.0011	
	square wave anodic-stripping voltammetry	thin mercury film	Cd(II)	white wine	set-up of the instrumental parameters and their optimization; environmental concerns related to the toxicity of mercury; UV photo-oxidative digestion of the sample	0 - 0.036	0.062	Illuminati et al., 2013
			Pb(II)			0 - 0.072	0.0058	
			Cu(II)			0 - 0.24	0.1	

	amperometry	poly(amidoamine) + Fe(II, III) oxide magnetic nanoparticles modified glassy carbon	bisphenol A	milk	no interferences from Na(I), Ca(II), Mg(II), Fe(III), Al(III), Zn(II), Cu(II), Cl(I); several pretreatment steps are required	0.0010 - 3.1	0.0050	Yin et al., 2011
	amperometry	molecular imprinted polymer + multi-walled carbon nanotubes and gold nanoparticles		honey grape juice	high selectivity; samples need to be filtered and diluted with acetonitrile	0.11 - 8200	0.0036	Huang et al., 2011
	cyclic voltammetry	hydroxy iron product + β-cyclodextrin on glassy carbon		beer	no matrix interferences (i.e. from redox active components); disadvantages not described	0.2 -10	0.50	Masikini et al., 2011
	linear sweep voltammetry	molecularly imprinted polymer microporous-metal-organic framework	tetracycline	honey	excellent selectivity; few pretreatment steps are required; preconditioning time 30 min.	2.2×10^{-7} - 0.022	2.2×10^{-10}	Bougrini et al., 2016
Electrochemical biosensors	electrochemical impedance spectroscopy	anti- acetamiprid aptamers + gold nanoparticles	acetamiprid	tomatoes	high selectivity; some pretreatment steps are required (homogenization, centrifugation and filtration); analysis time 3 h	0.005 - 0.6	0.001	Fan et al., 2013
	amperometry	transferrin + single-walled carbonnanotubes	Fe(III)	wine	no Zn(II) interferences; short analysis time (15 min); disadvantages not described	0.00089 - 0.036	0.00089	Cámara-Martos et al., 2016
	electrochemical impedance spectroscopy	anti- ochratoxin A aptamers + polythionine and iridium oxide nanoparticles carbon modified	ochratoxin A	wine	excellent specificity; disadvantages not described	1×10^{-5} - 0.1	1.4×10^{-7}	Rivas et al., 2015
	amperometry	diamine oxidase	biogenic amines	red and white wine beer	only sample dilution is required; less efficient in beer samples	7.9 - 230	2.3	Fusco et al., 2011

	amperometry	acetylcholin-esterase + iron oxide nanoparticles and carboxylated multi walled carbon nanotubes	malathion	milk	fast time response (4 s); no interferences from ascorbic acid and uric acid; inhibition effects of Hg(II) and Cr(VI) on enzymatic activity	0.0001 - 0.04	0.0001	Chauhan and Pundir, 2011
			chlorpyrifos			0.0001 - 0.05	0.0001	
			monocrotophos			0.0001- 0.05	0.001	
			endosulfan			0.01 - 0.1	0.01	
Optical Biosensors	fluorimetry	anti-aflatoxin M_1 antibodies labelled with fluorescein	aflatoxin M_1	milk	no interferences in the presence of aflatoxin B_1 and ochratoxin A; centrifugation of samples is needed	7.8×10^{-5} - 0.0062	1.6×10^{-5}	Sharma et al., 2016
	surface plasmon resonance	anti-chlorothalonil antibodies	chlorothalonil	vegetables (lettuce, cabbage, Welsh onion, cucumber eggplant)	sensitivity and the cross-reactivity were less effective than with the IC-ELISA; several pretreatment steps are required	0.0030 - 0.17	/	Hirakawa et al., 2015
	surface plasmon resonance	anti-triazines antibodies	atrazine simazine atrazinedesethyl azinphos-ethyl	milk	no sample pre-treatment are needed; shorter analysis time; much easier to perform (compared to amperometric and screen-printed devices); no substantial advantages (compared to amperometric and screen-printed devices)	0.1 -1.5	0.05	Tomassetti et al., 2015

	surface plasmon resonance	anti-melamine antibodies	melamine	infant formula	short analysis time (15 min); centrifugation of samples is necessary to remove fat	0 - 240	0.16	Wu et al., 2013
	surface plasmon resonance	anti-antibiotics antibodies	enrofloxacin<br>sulfapyridine<br>chloramphenicol	milk	no clean-up steps of samples are required; detection of chloramphenicol is slightly compromised by the sample dilution	0 - 2.8<br>0 - 2.0<br>0 - 2.2	8.3×10^{-7}<br>1.2×10^{-6}<br>8.0×10^{-7}	Fernández et al., 2010

REFERENCES

Bougrini M, Florea A, Cristea C, Sandulescu R, Vocanson F, Errachid A, et al. Development of a novel sensitive molecularly imprinted polymer sensor based on electropolymerization of a microporous-metal-organic framework for tetracycline detection in honey. *Food Control* (2016) 59:424–9. doi:10.1016/j.foodcont.2015.06.002

Cámara-Martos F, da Costa J, Justino CI, Cardoso S, Duarte AC, Rocha-Santos T. Disposable biosensor for detection of iron (III) in wines. *Talanta* (2016) 154:80–4. doi:10.1016/j.talanta.2016.03.057

Chauhan N, Pundir CS. An amperometric biosensor based on acetylcholinesterase immobilized onto iron oxide nanoparticles/multi-walled carbon nanotubes modified gold electrode for measurement of organophosphorus insecticides. *Anal Chim Acta* (2011) 701(1):66–74. doi:10.1016/j.aca.2011.06.014

Fan L, Zhao G, Shi H, Liu M, Li Z. A highly selective electrochemical impedance spectroscopy-based aptasensor for sensitive detection of acetamiprid. *Biosens Bioelectron* (2013) 43:12–8. doi:10.1016/j.bios.2012.11.033

Fernández F, Hegnerová K, Piliarik M, Sanchez-Baeza F, Homola J, Marco MP. A label-free and portable multichannel surface plasmon resonance immunosensors for on site analysis of antibiotics in milk samples. *Biosens Bioelectron* (2010) 26(4):1231–8. doi:10.1016/j.bios.2010.06.012

Fusco M, Federico R, Boffi A, Macone A, Favero G, Mazzei F. Characterization and application of a diamine oxidase from as component of an electrochemical biosensor for the determination of biogenic amines in wine and beer. *Anal Bioanal Chem* (2011) 2(401):707–16. doi:10.1007/s00216-011-5131-z

Hirakawa Y, Yamasaki T, Watanabe E, Okazaki F, Murakami-Yamaguchi Y, Oda M, et al. Development of an immunosensor for determination of the fungicide chlorothalonil in vegetables, using surface plasmon resonance. *J Agric Food Chem* (2015) 63(28):6325–30. doi:10.1021/acs.jafc.5b01980

Huang J, Zhang X, Lin Q, He X, Xing X, Huai H, et al. Electrochemical sensor based on imprinted sol-gel and nanomaterials for sensitive determination of bisphenol A. *Food Control* (2011) 22(5):786–91. doi:10.1016/j.foodcont.2010.11.017

Illuminati S, Annibaldi A, Truzzi C, Finale C, Scarponi G. Square-wave anodic- stripping voltammetric determination of Cd, Pb and Cu in wine: set-up and optimization of sample pre-treatment and instrumental parameters. *Electrochim Acta* (2013) 104:148–61. doi:10.1016/j.electacta.2013.04.001

Masikini M, Waryo TT, Baker PG, Ngqongwa LV, Williams AR, Iwuoha EI. Hydroxy-iron/β-cyclodextrin-film amperometric sensor for the endocrine disruptor substance bisphenol-A in an aqueous medium with reduced fouling effects. *Anal Lett* (2011) 44(11):2047–60. doi:10.1080/00032719.2010.539741

Rivas L, Mayorga-Martinez CC, Quesada-González D, Zamora-Gálvez A, de la Escosura-Muñiz A, Merkoçi A. Label-free impedimetric aptasensor for ochratoxin-A detection using iridium oxide nanoparticles. *Anal Chem* (2015) 87(10):5167–72. doi:10.1021/acs.analchem.5b00890

Shahbazi Y, Ahmadi F, Fakhari F. Voltammetric determination of Pb, Cd, Zn, Cu and Se in milk and dairy products collected from Iran: an emphasis on permissible limits and risk assessment of exposure to heavy metals. *Food Chem* (2016) 192:1060–7. doi:10.1016/j.foodchem.2015.07.123

Sharma A, Catanante G, Hayat A, Istamboulie G, Rejeb IB, Bhand S, et al. Development of structure switching aptamer assay for detection of aflatoxin M_1 in milk sample. *Talanta* (2016) 158:35–41. doi:10.1016/j.talanta.2016.05.043

Tomassetti M, Martini E, Campanella L, Favero G, Sanzó G, Mazzei F. A new surface plasmon resonance immunosensor for triazine pesticide determination in bovine milk: a comparison with conventional amperometric and screen-printed immunodevices. *Sensors (Basel)* (2015) 15(5):10255–70.doi:10.3390/s150510255

Wu H, Li H, Chua FZ, Li SFY. Rapid detection of melamine based on immunoassay using portable surface plasmon resonance biosensor. *Sens Actuators B Chem* (2013) 178:541–6. doi:10.1016/j.snb.2012.12.089

Yin H, Cui L, Chen Q, Shi W, Ai S, Zhu L, et al. Amperometric determination of bisphenol A in milk using PAMAM–Fe_3O_4 modified glassy carbon electrode. *Food Chem* (2011) 125(3):1097–103. doi:10.1016/j.foodchem.2010.09.098
